# Long-term outcomes of allogeneic stem cell transplant in multiple myeloma

**DOI:** 10.1038/s41408-023-00900-z

**Published:** 2023-08-18

**Authors:** Walker M. Schmidt, Nirosha D. Perera, Francis K. Buadi, Suzanne R. Hayman, Shaji K. Kumar, Angela Dispenzieri, David Dingli, Joselle Cook, Martha Q. Lacy, Prashant Kapoor, Nelson Leung, Eli Muchtar, Rahma M. Warsame, Taxiarchis Kourelis, Moritz Binder, Wilson I. Gonsalves, William J. Hogan, Morie A. Gertz

**Affiliations:** 1 Alix School of Medicine, Mayo Clinic, Rochester, MN USA; 2 Department of Internal Medicine, Mayo Clinic, Rochester, MN USA; 3 Department of Internal Medicine, Division of Hematology, Mayo Clinic, Rochester, MN USA; 4 Department of Internal Medicine, Division of Nephrology, Mayo Clinic, Rochester, MN USA

**Keywords:** Myeloma, Myeloma

## Abstract

Allogeneic stem cell transplant (allo SCT) for multiple myeloma (MM) is potentially curative in some, while toxic in many others. We retrospectively analyzed 85 patients diagnosed with MM who underwent allo SCT as frontline or salvage therapy between 2000 and 2022 at Mayo Clinic Rochester and examined patient outcomes and prognostic markers. Overall survival (OS), progression free survival (PFS), treatment related mortality (TRM), and relapse rates (RR) were estimated using the Kaplan Meier method and competing risk models. Median follow-up was 11.5 years. Median OS and PFS were 1.7 and 0.71 years, respectively. Five-year OS and PFS were 22.2% and 15.1%, respectively. One-year TRM was 23.5%. Twelve patients demonstrated durable overall survival, living 10+ years beyond their allo SCT. This subgroup was more likely to have no or one prior auto SCT (*p* = 0.03) and to have been transplanted between 2000 and 2010 (*p* = 0.03). Outcomes were poor in this cohort with long follow-up, with few patients surviving 5 years or more, and most relapsing or dying within 2 years. We would expect better outcomes and tolerability with an expanded array of novel therapeutics and would prefer them to allo SCT.

## Introduction

Multiple myeloma (MM) remains a largely incurable, clonal plasma cell disorder characterized by the presence of a monoclonal protein causing anemia, bone disease, and renal insufficiency. MM represents 1.8% of all new cancers in the United States; the median age at diagnosis is 70 years and the number of patients diagnosed annually is expected to double over the next 20 years [1]. With this increasing burden of disease, identifying an expanded set of treatment options is paramount. Initial treatment for MM depends on the patient’s eligibility for autologous stem cell transplant (auto SCT), as determined by disease stratification and patient functional status. Transplant-eligible patients often receive induction chemotherapy followed by autologous stem cell mobilization, harvest, and transplant. Transplant ineligible patients will commonly receive a variety of immunomodulatory drugs (ImID) and proteosome inhibitor-based regimens. Novel agents such as the proteasome inhibitors, in addition to monoclonal antibodies, chimeric antigen receptor T (CAR-T) cells, and bispecific T cell engagers (BiTE) have shown promising results but are not available in many countries [2, 3].

The use of allogeneic stem cell transplant (allo SCT) is controversial. For some, it offers a potentially curative modality due to a graft vs myeloma effect [4]. For many, it incurs considerable toxicity due to graft-vs-host disease (GVHD) [5]. For this reason, allo SCT is not routinely offered in MM patients, although utilization has increased over the past 30 years despite a lack of clear treatment guidelines [6, 7]. Today, Allo-SCT is generally reserved for young patients with high risk, relapsed MM [8], and has mixed efficacy overall [9–12]. With the development of modern novel therapies in the last decade, the role of allo SCT is increasingly difficult to define.

Our project aims to characterize patient outcomes and prognostic markers in patients who received allo SCT. With a median follow up of 11.5 years in a relatively large cohort of patients with multiple myeloma, we identify patients with prolonged favorable outcomes after allo SCT, which previous studies with shorter follow-up could not detect. A greater understanding of allo SCT patient outcomes and prognostic factors can help guide treatment decisions for this potentially life-prolonging and perhaps even curative therapy.

## Methods

### Patients’ description and data source

We retrospectively analyzed patients diagnosed with MM who underwent allo SCT between 2000 and 2022 at Mayo Clinic Rochester. Data were analyzed as of May 2022; patient data were retrieved from our institution’s continuously maintained database and patient electronic medical records. Patients received regular clinical follow-up. The analysis was conducted according to the Declaration of Helsinki and Good Clinical Practice guidelines. All patients provided written informed consent for institution-initiated research studies. This project was approved by Mayo Clinic’s Institutional Review Board.

High risk (HR) cytogenetics were defined as the presence of del17p, t(4;14), or t(14;16) [13] and 1q+ at the time of diagnosis [14]. There was not enough available data regarding cytogenetics at the time of diagnosis or S-phase fraction to perform statistical analysis. Conditioning regimen intensities and graft-versus-host disease (GVHD) were categorized according to previously published criteria [15, 16]. The presence of chronic GVHD (cGVHD) was determined according to the 2014 NIH GVHD criteria [17]. The number of prior lines of therapy were determined according to previously proposed guidelines [18]. The International Myeloma Working Group (IMWG) criteria were used to define MM disease and response status [19]. The primary endpoints were overall survival (OS) and progression-free survival (PFS).

### Statistical analyses

All data were analyzed using BlueSky Statistics software version 7.4 (BlueSky Statistics LLC, Chicago, IL, USA). Descriptive statistics used the median for continuous variables and counts and percentages for categorical variables. The patient, treatment, and disease characteristics for those surviving less than 10 years ( <10 yr.) versus those surviving more than 10 years (10+ yr.) were compared for independence, and p values were generated using χ2 statistics for categorical variables and the Mann-Whitney test with a two-tailed hypothesis for continuous variables. OS and PFS were calculated as the time from allo SCT to death from any cause, and the first observation of relapse or death from any cause, respectively. Treatment-related mortality (TRM) was defined as death secondary to transplant complications without progressive disease. Patients without observation of the event of interest at the last follow-up were censored. OS and PFS rates were estimated and reported using the Kaplan-Meier method. Relapse/progression and TRM were considered to be competing risks and estimated as cumulative incidence rates using Aalen Johansen estimator [20] and compared with Fine and Gray regression models for competing risks [21], similarly to previously published studies [22]. For subgroup analysis, if 5-year OS or PFS of a variable was not available, 5-year survival was estimated using the value of the nearest timepoint. Univariate analysis was conducted using the Cox proportional hazards model.

## Results

Information on 91 patients with multiple myeloma who underwent an allo SCT between 2000 and 2022 was available in our institution’s database. Six patients were excluded for having undergone allo SCT in the setting of a myelodysplastic disorder secondary to previous MM treatment, not as primary treatment for MM, leaving 85 patients available for final analysis. Baseline patient characteristics are summarized in Table 1. Median age at the time of allo SCT was 51.2 years and median time from diagnosis of MM to allo-SCT was 2.7 years. By sex, 72.6% (*N* = 61) were male and 27.4% (*N* = 24) female. Most participants were White (89.4%, *N* = 76). Eighty-one patients (95.3%) had undergone one or more auto SCTs, while four (4.7%) had no previous auto SCT. Most patients (56.5%, *N* = 48) received their allo SCT two to five years from the time of MM diagnosis. Among those with a prior auto SCT, most patients received their allo SCT within two years of their auto SCT (55.6%, *N* = 45). The cytogenetic risk profile at time of diagnosis was available for 94.1% of participants (*N* = 80), with 66.3% (*N* = 53) of patients categorized as standard risk and 33.8% (*N* = 27) of patients categorized as high risk. Approximately half of patients (*N* = 47, 55.3%) received a myeloablative (MA) conditioning regimen in preparation for allo SCT, while the rest (*N* = 38, 44.7%) received a reduced-intensity (RIC) or nonmyeloablative (NMA) regimen. Patients underwent a median of 4 prior lines of therapy before allo SCT. Median follow-up for the cohort was 11.5 years.

**Table 1:** Patient, treatment, and disease characteristics.

	Overall Cohort (N)	<10 yr. survival	10+ yr. survival	*P* value (<10 yr. vs 10+ yr. survival)
Patients	85	73	12	
Age				0.69
<50	38	32	6	
50+	47	41	6	
Sex				0.07
Male	61	52	9	
Female	24	21	3	
Race				0.11
White	76	65	11	
Unknown	4	4	0	
Other	3	3	0	
Black or African American	1	1	0	
Asian Chinese	1	0	1	
Prior Auto SCT(s)				0.03*
0	4	2	2	
1	63	53	10	
2	18	18	0	
Years from MM Dx to Allo SCT				0.54
0–2	20	17	3	
2–5	48	40	8	
5+	17	16	1	
Years from Auto SCT_1_ to Allo SCT	*N* = 81	*N* = 71	*N* = 10	0.33
0–2 years	45	38	7	
2+ years	36	33	3	
Auto SCT_1_ Response at Day 100	N = 70	N = 60	N = 10	0.39
CR	30	26	4	
VGPR	10	7	3	
PR	25	23	2	
Progression	5	4	1	
Years from Auto SCT_2_ to Allo SCT	(*N* = 18)	*N* = 18	*N* = 0	—
0–2 years	14	14	—	
2+ years	4	4	—	
Auto SCT_2_ Response at Day 100	(*N* = 10)	(*N* = 9)	(*N* = 1)	0.86
CR	1	1	0	
VGPR	1	1	0	
PR	6	5	1	
Progression	2	2	0	
Allo Date				0.03*
2000–2010	46	36	10	
2011-	39	37	2	
Response Status Going Into Allo SCT (*N* = 78)				0.58
CR	14	10	4	
VGPR	11	10	1	
PR	22	21	1	
Stable Disease	7	5	2	
Progression	24	22	2	
Cytogenetic Risk (*N* = 80)				0.17
Standard	53	43	10	
High risk	27	25	2	
Transplant Intensity				0.21
MA	47	38	9	
RIC	26	23	3	
NMA	12	12	0	
Transplant course:				
Standalone MA allo SCT	47	38	9	
Standalone RIC allo SCT	15	14	1	
Standalone NMA allo SCT	6	6	0	
Upfront auto SCT to RIC allo SCT	3	3	0	
Auto SCT to RIC (or NMA) allo SCT for relapse	14	12	2	
Acute GVHD				0.97
Present	50	43	7	
Absent	35	30	5	
Chronic GVHD				0.69
Present	38	32	6	
Absent	47	41	6	
Lines of Therapy Before Allo SCT				0.17
1–3	21	18	3	
4–6	48	39	9	
7+	16	16	0	
Donor Type	(*N* = 84)			0.94
PBSC Related	50	43	7	
PBSC Unrelated	32	27	5	
Single UC Unrelated	1	1	0	
Double UC Unrelated	1	1	0	
Median (years)				
Age at allo transplant	51.2	51.2	50.3	0.71
Time from MM diagnosis to allo SCT	2.65	2.77	2.48	0.22
Time from auto SCT_1_ to allo SCT	1.88	1.88	1.69	0.15
Time from auto SCT_2_ to allo SCT	0.54	0.54	—	—
Lines of therapy prior to Allo SCT	4	4	4	—
OS	1.86	1.14	13.0	<0.01*
PFS	0.71	0.59	10.4	<0.01*
1-year treatment-related mortality (TRM)	20	20	—	—

*Auto SCT* autologous stem cell transplant.

*Allo SCT* allogeneic stem cell transplant.

*CR* complete response.

*VGPR* very good partial response.

*PR* partial response.

*MA* myeloablative.

*RIC* reduced-intensity chemotherapy.

*NMA* nonmyeloablative.

*GVHD* graft-versus host disease.

*PBSC* peripheral blood stem cell.

*UC* umbilical cord.

*p < 0.05.

Bold entries denote statistically significant *p* values.

**Fig. 1:**
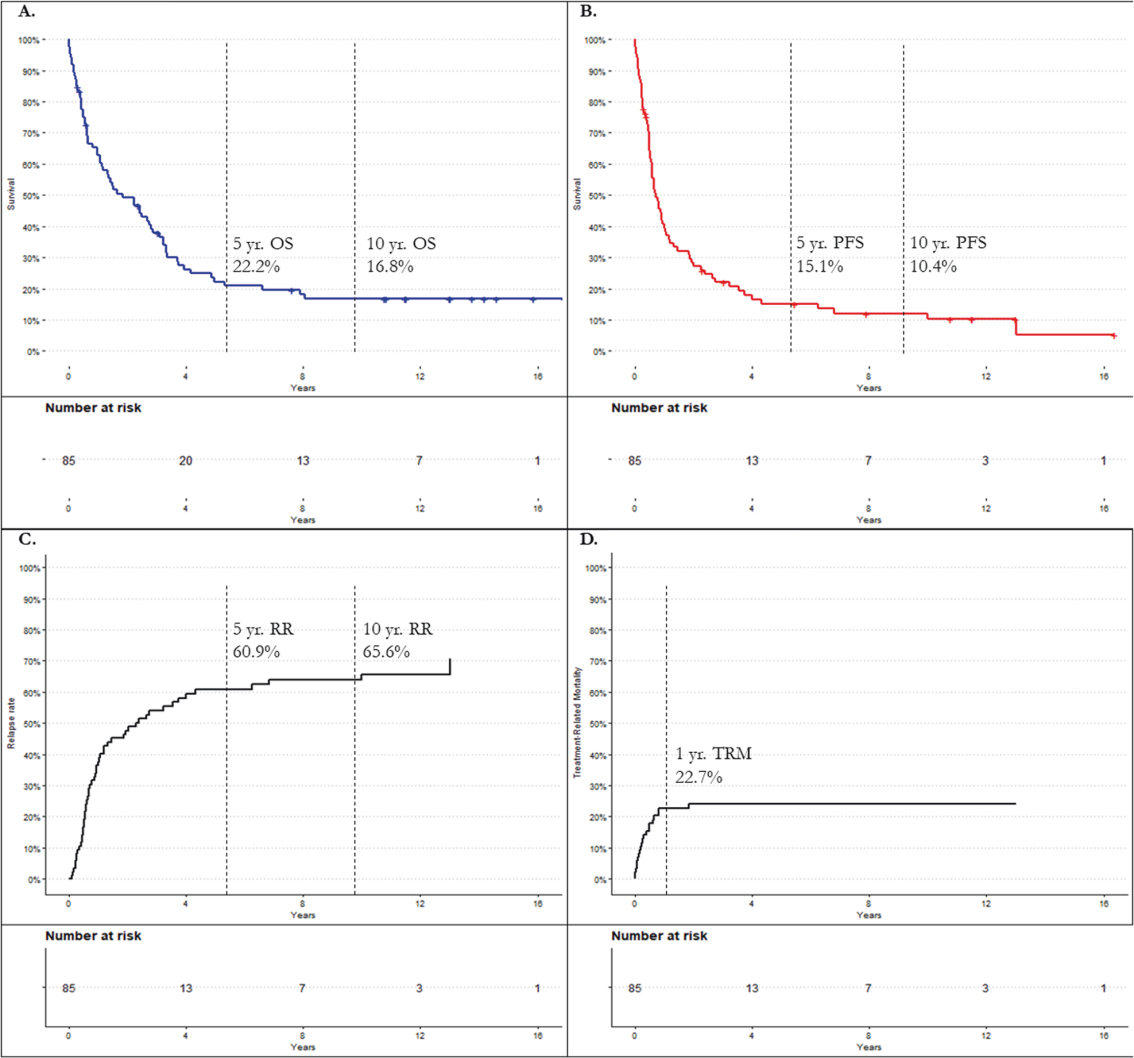
Outcome analysis of the entire cohort. **A** Kaplan-Meier estimates for overall survival (OS). **B** Kaplan-Meier estimates for progression-free survival (PFS). **C** Cumulative incidence of relapse rate (RR). **D** Cumulative incidence of treatment-related mortality (TRM). allo-SCT allogeneic stem cell transplantation, yr. year.

Median OS for the entire cohort was 1.7 years. 3-year OS was 37.9%, 5-year OS was 22.2%, and 10-year OS was 16.8%. Median PFS in this cohort was 0.71 years. 3-year PFS was 22.0%, 5-year PFS was 15.1%, and 10-year PFS was 10.4% (Fig. 1). TRM at 1-year post-allo transplant was 23.5%. At the time of data analysis in May 2022, 18 patients (21.2%) were alive. 5-year OS and PFS were stratified by the various patient, disease, and treatment characteristics as described above. These univariate analysis findings are outlined in Table 2. Briefly, outcome in this cohort was not significantly impacted by age, race, baseline cytogenetic risk, myeloablative regimen, or timing of allo SCT, among others. Active progression, compared to those in a complete remission at the time of allo SCT arose as a significant prognostic factor of OS (HR 3.39, *p* = <0.01). Development of chronic GVHD (cGVHD), (*p* = 0.04) and number of prior auto SCT’s, (*p* = 0.02) also arose as prognostic factors for OS, though some subgroups were small. Prognostic factors for PFS followed a similar pattern, with progression at the time of allo-SCT arising as the most prominent negative prognostic factor (HR 4.39, *p* = <0.01). The number of prior auto SCTs was also a prognostic factor (*p* = 0.02).

**Table 2:** Univariate analysis of OS and PFS by patient, treatment, and disease characteristics.

	N	5-yr OS %	OS HR (95% CI, *p* = )	5-yr PFS %	PFS HR (95% CI, *p* = )
Entire Cohort	85	22.2	N/A	15.1	—
Age					
<50	38	27.9	Ref.	15.8	Ref.
50+	47	18.0	1.36 (0.37–2.21, p = 0.22)	12.1	1.33 (0.84–2.12, *p* = 0.23)
Sex					
Male	61	22.6	Ref.	16.1	Ref.
Female	24	20.8	1.26 (0.75–2.11, *p* = 0.38)	12.5	1.27 (0.77–2.11, *p* = 0.34)
Race					
White	76	22.1	—	16.9	—
Unknown	4	0	—	0	—
Other	3	0	—	0	—
Black or African American	1	0	—	0	—
Asian Chinese	1	100	—	0	—
Prior Auto SCT(s)					
0	4	50.0	Ref.	0.75	Ref.
1	63	24.4	2.62 (0.63–10.83, *p* = 0.18)	0.14	2.49 (0.76–8.16, *p* = 0.13)
2	18	**0**	**5.68** (1.28–25.22, *p* = 0.02*)	0	**4.86 (1.36–17.39,** ***p*** = 0.02*)
Years MM Dx to Allo SCT					
0–2	20	30.0	Ref.	18.7	Ref.
2–5	48	19.8	1.24 (0.68–2.23, *p* = 0.47)	12.9	1.39 (0.78–2.48, *p* = 0.27)
5+	17	18.1	1.28 (0.61–2.66, *p* = 0.51)	9.6	1.44 (0.71–2.93, *p* = 0.31)
Years Auto SCT_1_ to Allo SCT (*N* = 81)					
0–2 years	45	20.0	Ref.	10.5	Ref.
2+ years	36	18.4	1.11 (0.68–1.82, *p* = 0.67)	10.8	1.15 (0.72–1.84, *p* = 0.57)
Auto SCT_1_ Response at Day 100 (*N* = 70)					
CR	30	29.2	Ref.	19.1	Ref.
VGPR	10	30.0	0.93 (0.39–2.18, *p* = 0.86)	20.0	0.79 (0.35–1.75, *p* = 0.55)
PR	25	12.0	**1.96 (1.08–3.56,** ***p*** = 0.03*)	8.5	1.30 (0.73–2.31, *p* = 0.36)
Progression	5	20.0	1.40 (0.39–2.18, *p* = 0.86)	0	1.31 (0.50–3.45, *p* = 0.58)
Years Auto SCT_2_ to Allo SCT (N = 18)					
0–2 years	14	0	Ref.	0	Ref.
2+ years	4	25	0.64 (0.18–2.27, *p* = 0.49)	25	0.40 (0.11–1.44, *p* = 0.16)
Auto SCT_2_ Response at Day 100 (*N* = 10)					
CR	1	0	Ref.	100	—
VGPR	1	0	0.71 (0.04–12.31, *p* = 0.82)	0	—
PR	6	33.3	0.33 (0.03–3.52, *p* = 0.36)	0	—
Progression	2	0	0.49 (0.03–7.10, *p* = 0.60)	0	—
Allo Date					
2000–2010	46	23.9	Ref.	14.6	Ref.
2011-	39	20.3	1.32 (0.82–2.15, *p* = 0.26)	16.0	1.27 (0.79–2.02, *p* = 0.32)
Response Status Going Into Allo SCT (*N* = 78)					
CR	14	42.9	Ref.	35.9	Ref.
VGPR	11	11.3	1.94 (0.74–5.07, *p* = 0.18)	9.1	2.41 (0.93–6.23, *p* = 0.06)
PR	22	21.2	1.81 (0.81–4.02, *p* = 0.15)	5.9	2.13 (0.93–4.88, *p* = 0.07)
Stable disease	7	28.6	1.35 (0.45–4.04, *p* = 0.59)	28.6	1.58 (0.54–4.62, *p* = 0.40)
Progression	24	10.4	**3.39 (1.54–7.46,** ***p*** = <0.01*)	4.2	**4.39 (1.93–9.98,** ***p*** = <0.01*)
Cytogenetic Risk (*N* = 80)					
Standard	53	22.1	Ref.	16.9	Ref.
High risk*	27	11.5	1.10 (0.66–1.82, *p* = 0.72)	10.2	1.35 (0.79–2.28, *p* = 0.26)
Transplant Intensity					
MA	47	28.3	Ref.	19.8	Ref.
RIC	26	20.1	0.93 (0.54–1.60, *p* = 0.79)	13.8	0.85 (0.50–1.45, *p* = 0.56)
NMA	12	8.3	1.18 (0.60–2.30, *p* = 0.63)	0	1.22 (0.63–2.37, *p* = 0.55)
Transplant course:					
Standalone MA allo SCT	47	28.3	Ref.	19.8	Ref.
Standalone RIC allo SCT	15	14.4	1.20 (0.63–2.27, *p* = 0.59)	7.2	1.04 (0.56–1.96, *p* = 0.90)
Standalone NMA allo SCT	6	0	1.22 (0.50–2.93, *p* = 0.66)	0	1.36 (0.57–3.23, *p* = 0.49)
Upfront auto SCT to RIC allo SCT	3	33.3	0.78 (0.24–2.55, *p* = 0.68)	0	0.86 (0.23–2.80, *p* = 0.80)
Auto SCT to RIC (or NMA) allo SCT for relapse	14	21.4	0.84 (0.42–1.65, *p* = 0.60)	19.1	0.78 (0.40–1.53, *p* = 0.47)
Acute GVHD					
Present	50	23.6	Ref.	11.7	Ref.
Absent	35	18.9	0.91 (0.56–1.49, *p* = 0.71)	19.9	0.93 (0.58–1.49, *p* = 0.75)
Chronic GVHD					
Present	38	23.1	Ref.	16.2	Ref.
Absent	47	18.0	**1.65 (1.02–2.71,** ***p*** = **0.04)**	14.2	1.34 (0.84–2.13, *p* = 0.22)
Lines of Therapy Before Allo SCT					
1–3	21	23.8	Ref.	12.9	Ref.
4–6	48	25.7	1.03 (0.57–1.81, *p* = 0.91)	19.3	1.05 (0.59–1.85, *p* = 0.87)
7+	16	0	1.50 (0.71–3.16, *p* = 0.28)	0	1.97 (0.98–3.97, *p* = 0.06)
Donor Type (*N* = 84)					
PBSC Related	50	21.0	Ref.	16.3	Ref.
PBSC Unrelated	32	22.3	0.89 (0.54–1.49, *p* = 0.67)	14.9	0.94 (0.58–1.53, *p* = 0.82)
Single UC Unrelated	1	0	1.01 (0.14–7.41, *p* = 0.99)	0	1.02 (0.14–7.57, *p* = 0.97)
Double UC Unrelated	1	0	**9.6** (1.19–77.46, *p* = 0.04*)	0	**8.61** (1.08–68.50, *p* = 0.04*)

*HR* Hazard Ratio.

*Ref.* Reference variable for hazard ratio generation.

*Auto SCT* autologous stem cell transplant.

*Allo SCT* allogeneic stem cell transplant.

*CR* complete response.

*VGPR* very good partial response.

*PR* partial response.

*MA* myeloablative.

*RIC* reduced-intensity chemotherapy.

*NMA* nonmyeloablative.

*GVHD* graft-versus host disease.

*PBSC* peripheral blood stem cell.

*UC* umbilical cord.

*p < 0.05.

Bold entries denote statistically significant *p* values.

Twelve patients (14%) demonstrated durable overall survival, living more than 10 years beyond their allo SCT. Comparison of patient, treatment, and disease characteristics among those surviving <10 yrs. versus 10+ yrs. are outlined in Table 1. The number of prior auto SCTs and date of allo transplant arose as statistically significant differences among these groups, with the 10+ yr. survivor subgroup being more likely to have no or one prior auto SCT (*p* = 0.03) and to have been transplanted between 2000 and 2010 (*p* = 0.03). No difference was found among other characteristics such as baseline cytogenetic risk, myeloablation regimen, or number of previous lines of therapy. Sex approached, but did not reach, statistical significance (*p* = 0.07), with a higher proportion of 10+ yr. survivors being male.

## Discussion

This study demonstrates overall poor survival outcomes in patients who underwent allo SCT for treatment of multiple myeloma with long-term survival ( >5 years) being achieved in less than 25% of patients. Nearly 75% of patients experienced disease progression or death by 2 years, and 1-year TRM was high at 22.2% Despite this, a subgroup of patients entered a durable remission after receiving an allo SCT, potentially constituting a cure. Those who saw particular benefit from allo SCT were more likely to have been in complete remission at time of allo SCT, had only one previous auto SCT, and to have undergone their allo transplant in the early 2000s. In all, allo-SCT as a treatment for multiple myeloma was minimally beneficial outside of a select few patients.

5-year OS following allogeneic transplant was poor at 22.2% with a cohort size of 85 patients and a long median follow-up of 11.5 years. As demonstrated by Fig. 1, there was a rapid survival decline immediately following transplant that did not plateau until roughly four years post-transplant and did not entirely stabilize until eight years post-transplant. 5-year PFS was 15.1%. Cumulative 1-year TRM was high at 23.5% (N = 20), exceeding the number of “cured” patients who survived 10+ years (N = 12). The largest study to examine allo SCT in MM was conducted by the Bone Marrow Transplant Clinical Trials Network (BMT CTN), and found 3-year OS and PFS to be 77% and 43%, respectively, with a TRM of 11% [23] for patients who underwent allo SCT as upfront therapy. Our overall cohort demonstrated considerably worse outcomes for the same timepoints with 3-year OS and PFS being 37.9% and 22.0%, respectively.

It is important to note that the findings of the BMT CTN study represent allogeneic transplant as upfront treatment (auto SCT to RIC allo SCT) in a group of individuals primarily with standard-risk cytogenetics and partial-response or better to previous therapy. Conversely our data represented a heterogenous and highly challenging population usually at a salvage point, with over 75% having at least 3 previous lines of therapy, and over 30% of patients in active progression at the time of transplant. The poorer survival outcomes in our study were not unexpected, given the prolonged disease history and more extensive prior therapies. Even when examining the few among our cohort who underwent auto SCT to RIC allo SCT as upfront therapy (N = 3), OS and PFS were 33% and 19%, respectively, considerably lower than the BMT CTN study.

As such, our findings more closely align with those from studies examining allo SCT as salvage therapy. Numerous studies have been conducted over the years with considerable variability in survival findings depending on pretransplant disease and patient characteristics, with estimated 2-year OS and PFS between 32–54% and 19–42%, respectively [12, 24, 25], and estimated 5-year OS and PFS between 14–26% and 2–20%, respectively [12, 24–27]. The largest allo SCT MM salvage study [28], with 413 patients, found a 5-year OS of 30% with a cumulative 1-year TRM of 21.5%, with no apparent plateau in the survival curves.

With a median follow-up of 11.5 years, our study provides unique insight into the long-term outcomes of allo SCT in MM patients. Extended follow-up revealed an OS plateau at eight years following allo SCT, with roughly 15% of patients (*N* = 12) surviving beyond 10 years, representing a potentially cured subgroup. Descriptive examination of patient and disease characteristics among this set of “long-term” survivors reveals them to have a higher proportion of individuals with no or one previous auto SCT, and allo SCT date between 2000 and 2010. Those receiving heavy pretreatment likely had a more aggressive or advanced disease at time of allo SCT. Regarding transplant dates, allo SCT was more commonly used as upfront therapy in the 2000s, whereas by the 2012 over two-thirds of allo SCT’s were performed as salvage therapy for progressive disease due to the advent and increased usage of novel therapeutics [7]. Of note, any patients in this study who underwent allo SCT in May 2012 or beyond had not been followed long-enough to reveal a 10-year survival at the time of analysis (May 2022). Six patients among the allo-SCT 2011-to-present analysis group were still alive at the time of analysis, five of whom underwent allo SCT from 2019 onward, and one who underwent allo SCT in 2014.

Finally, among the entire cohort, univariate analysis for prognostic factors in survival showed patients with disease progression at the time of allo SCT did poorly, the presence of chronic GVHD conferred a survival benefit, and the presence of two prior auto SCT’s conferred a survival detriment. Though this could represent the previously described survival advantage of mild cGVHD [6], it may indicate survival bias toward a subset of patients who survived long enough to develop cGVHD. Our study’s finding that two prior auto SCT’s is associated with worsened OS and PFS likely reflects the trend that patients who are heavily pre-treated, in general, have more aggressive or advanced disease and are at higher risk of mortality.

This study should be interpreted in the context of several strengths and limitations. The cohort was quite racially/ethnically homogenous with most participants identifying as White. Future studies should aim to include a more diverse and ethnically representative cohort. Furthermore, small numbers among some patient characteristic subgroups limited the power of statistical analyses. Notwithstanding these limitations, the long median follow-up of this study allowed for examination of the extended outcomes and the ability to rely on the higher accuracy of observed, rather than estimated, OS and PFS. Additionally, the cohort size of 85 provides a relatively large series among these patients [29].

In conclusion, allogeneic transplant poses a therapeutic dilemma for myeloma clinicians. Particularly as salvage therapy, allo SCT has poor outcomes. It is curative for a small subset of patients, however, the patient, treatment, and disease characteristics that predispose those in this subgroup to favorable long-term outcomes remain ill-defined. Unfortunately, the curative potential of allo SCT is tempered by poor OS, poor PFS, and a high TRM that exceeded the “cure” rate among the rest of the observed patients. The benefit of allo SCT may be tremendously high—a potential cure—but the risk is even higher. With an expanding array of anti-CD38, BiTE, and other novel therapeutics, we would expect better overall outcomes and tolerability of these agents compared to allo SCT and, if available, would prefer them to allo SCT.

## Data Availability

The data that support the findings of this study are available from the corresponding author (WMS) upon reasonable request.
